# Investigating the relationship between digital citizenship levels and cyberbullying attitudes of university students

**DOI:** 10.3389/fpsyg.2025.1664397

**Published:** 2025-10-29

**Authors:** Metin Karayol, Talha Murathan, Ramazan Erdoğan, Mehmet Akarsu, Muhammet Baş, Göktuğ Norman

**Affiliations:** ^1^Department of Sport Management, Faculty of Sport Sciences, Mus Alparslan University, Mus, Türkiye; ^2^Department of Sport Management, Faculty of Sport Sciences, Inonu University, Malatya, Türkiye; ^3^Department of Coach Education, Faculty of Sport Sciences, Munzur University, Tunceli, Türkiye; ^4^Department of Physical Education and Sports, Faculty of Sport Sciences, Mus Alparslan University, Mus, Türkiye

**Keywords:** physical education and sports, digital citizenship, cyberbullying, internet security, use of technology

## Abstract

**Background:**

This study examines the relationship between the levels of digital citizenship and attitudes toward cyberbullying among prospective physical education and sports teachers.

**Methods:**

This quantitative study adopted the relational survey model within its research framework. The sample consists of prospective physical education and sports teachers who studied in the Physical Education and Sports Teaching departments at universities during the 2022–23 academic year. The sample was determined using the convenience sampling method. The sample was administered a personal information form created by the researchers, as well as the Digital Citizenship Scale and the Cyberbullying Attitude Scale.

**Results:**

According to the MANOVA results for the Digital Citizenship and Cyberbullying Attitude scales, significant differences were found in the Digital Law, Digital Rights and Responsibilities, Approval and Anxiety subscales based on gender. However, the Pearson correlation analysis revealed no significant relationship between age and the subscales of the Digital Citizenship and Cyberbullying Attitude scales. However, the Pearson correlation analysis of the Digital Citizenship and Cyberbullying Attitude subscales revealed significant positive and negative relationships.

**Conclusion:**

The results of the path analysis of the digital citizenship scale and its subscales (identity concealment, enjoyment, approval and anxiety) showed that digital citizenship significantly predicted identity concealment, enjoyment, approval and anxiety.

## 1 Introduction

Research in this area is limited, but it is important to note that cyberbullying can also occur in sports and physical education settings. The extant literature suggests that cyberbullying can have negative psychosocial impacts on athletes and students. In order to develop effective prevention and intervention strategies in these settings, there is a need for greater conceptual and methodological clarity. While the prevalence of cyberbullying in the school physical education context is influenced by factors such as age, school year, and access to technology, gender appears to be less of a determining factor (MacPherson and Kerr, 2023; Köroǧlu et al., 2025). It is evident that a multitude of phenomena across the globe have evolved into multidimensional structures, a development that has been precipitated by the rapid advancement of information and communication technologies. Among these, the internet is particularly noteworthy due to its numerous advantages, including opportunities for learning, teaching, communication, employment, entertainment, access to information, and social solidarity. However, it is important to note that when internet use is not deliberate or when adequate precautions are not followed, there are significant risks associated with this practice. The prevalence of online games, entertainment platforms and social networks has given rise to a number of issues, including violations of personal privacy and cyberbullying. These issues have the potential to result in legal complications by infringing on individuals’ privacy and causing lasting harm to their rights and freedoms. Recent technological developments in the domain of information and communication technologies have precipitated a considerable transfer of real-life activities, such as education, commerce and communication, into digital environments. This paradigm shift has given rise to the notion of digital citizenship, which aims to safeguard the rights and liberties of individuals and institutions within digital spaces through legal mechanisms (Kocatürk, 2014). The notion of digital citizenship was initially conceptualised by Ribble and Bailey (2004), who defined it as a set of behavioural norms pertaining to the utilisation of technology. Ribble (2008) further described it as the reflection of effective, safe and responsible use of technology on citizenship. Mossberger (2009) defined “digital citizenship” as the capacity to participate in online social activities and to utilise information technology effectively. In contrast, Chadwick and Howard (2009) highlighted the political and social impacts of the Internet. In a similar vein, Gencer (2017), İşman and Güngören (2013) ve Yelci (2018) have emphasised the role of the internet in education, while Kaptangil and Çalışır (2023) have addressed the challenges currently being faced. Castells (2000) highlighted the internet as a defining element of the network society, with individuals in this context identified as digital citizens.

It is evident that technological advancements have been advantageous in many respects. However, it must be noted that the concomitant proliferation of digital tools has also given rise to a number of new challenges. Of these issues, one of the most pressing is that of cyberbullying. Cyberbullying is defined as the intentional, repetitive, and harmful behaviour of an individual or group directed at a more vulnerable person through digital tools (Smith et al., 2008; Pekşen Süslü, 2016). Those exposed to such behaviors are defined as cyber victims. A study conducted with university students in China found that cyberbullying is low-level but influenced by individual factors such as gender, personality traits, life satisfaction, empathy, and digital citizenship. Digital citizenship skills and legal awareness have been demonstrated to be effective in reducing cyberbullying, while internet addiction and inadequate online communication skills have been shown to increase the risk (Zhong et al., 2021).

It is well documented that individuals who have been subjected to victimisation, particularly in the context of cyberbullying, are prone to a range of adverse psychological, emotional and social consequences. Exposure to cyberbullying has been demonstrated to be associated with elevated levels of depression and anxiety, which can exert a substantial influence on overall mental health and daily functioning (Prıce and Dalgleısh, 2010). As is well documented, victims frequently experience a decline in self-confidence and self-esteem, which can result in feelings of inadequacy and social withdrawal. In some cases, prolonged exposure has been demonstrated to be a contributing factor to the emergence of clinical anxiety disorders and increased stress responses. Moreover, research has demonstrated that cyberbullying has the capacity to evoke profound feelings of anger and a strong desire for retribution. In extreme cases, it has been observed to lead to suicidal thoughts. The consequences for victims of such experiences are frequently reported to include feelings of loneliness, social isolation and a general sense of dissatisfaction with life. These factors have the potential to hinder academic performance, professional development, and interpersonal relationships. The collective impact of cyber victimisation has been demonstrated to be significant, affecting numerous areas of an individual’s life and well-being (Raskauskas and Stolz, 2007; Topçu et al., 2008; Perren et al., 2012; Olenık Shemesh et al., 2012; Türkileri et al., 2013). As demonstrated in the works of Bonanno and Hymel (2013), Prıce et al. (2013), Dolgın (2014), Brewer and Kerslake (2015), Baruah et al. (2017), Chu et al. (2018), Tıan et al. (2018), and Yurdakul (2020).

Cyberbullying is regarded as a substantial problem among Turkish adults, particularly university students, where platforms such as Instagram and TikTok are extensively utilised (Şengül, 2024). The most prevalent forms of cyberbullying include offensive comments, hate speech, and novel tactics such as emoji-based mocking. Research indicates that both cyberbullying and victimisation are prevalent phenomena, with a moderate correlation observed between victimisation and perpetration. A preponderance of research has identified a higher propensity among males to engage in both the perpetration and victimisation of cyberbullying (see Akbulut and Erişti, 2011 for a review). The aetiology of this behaviour is often attributed to interpersonal challenges experienced by the perpetrators (ibid). The advent of the internet has had a profound impact on university students, with the increase in its use giving rise to a number of problematic behaviors. Among these is the phenomenon of cyberbullying and online harassment, which has become increasingly prevalent. The impact on learning skills of a negative nature can be exacerbated by emotional issues such as anxiety, depression and loneliness. Research indicates that the integration of digital citizenship behaviors into educational environments can serve as a mitigating factor against the aforementioned negative effects. Furthermore, the relationship between digital citizenship and cyberbullying is found to be indirectly influenced by perceived learning outcomes (Dunaway and Macharia, 2021). The extant literature highlights the prevalence of cyberbullying as a significant problem among university students, with particular reference to social media platforms. Research findings indicate the prevalence of various forms of bullying, including offensive comments, hate speech, and emoji-based mocking. Students’ responses to these situations vary, ranging from passive bystanders to active intervention. The findings emphasise the significance of educational programmes, institutional mechanisms, and peer support networks that promote digital citizenship. They also indicate that awareness-raising initiatives in university settings can be efficacious in addressing cyberbullying (Şengül, 2024).

Prospective physical education and sports teachers graduate from teacher training programmes within the faculties of sports sciences and colleges of universities, pass professional and central examinations, and begin their teaching careers. The institution’s comprehensive training programme, complemented by its emphasis on digital citizenship and the prevention of cyberbullying, has a positive impact on its future student body. In the context of this study, the objective was to examine the relationship between digital citizenship levels and cyberbullying tendencies of prospective physical education and sports teachers. This was achieved by assessing both their digital citizenship levels and their cyberbullying attitudes.

## 2 Materials and methods

### 2.1 Participants

This section comprises information regarding the research model, population and sample, data collection tools, data collection process, and data analysis steps of the study.

**Figure 1:**
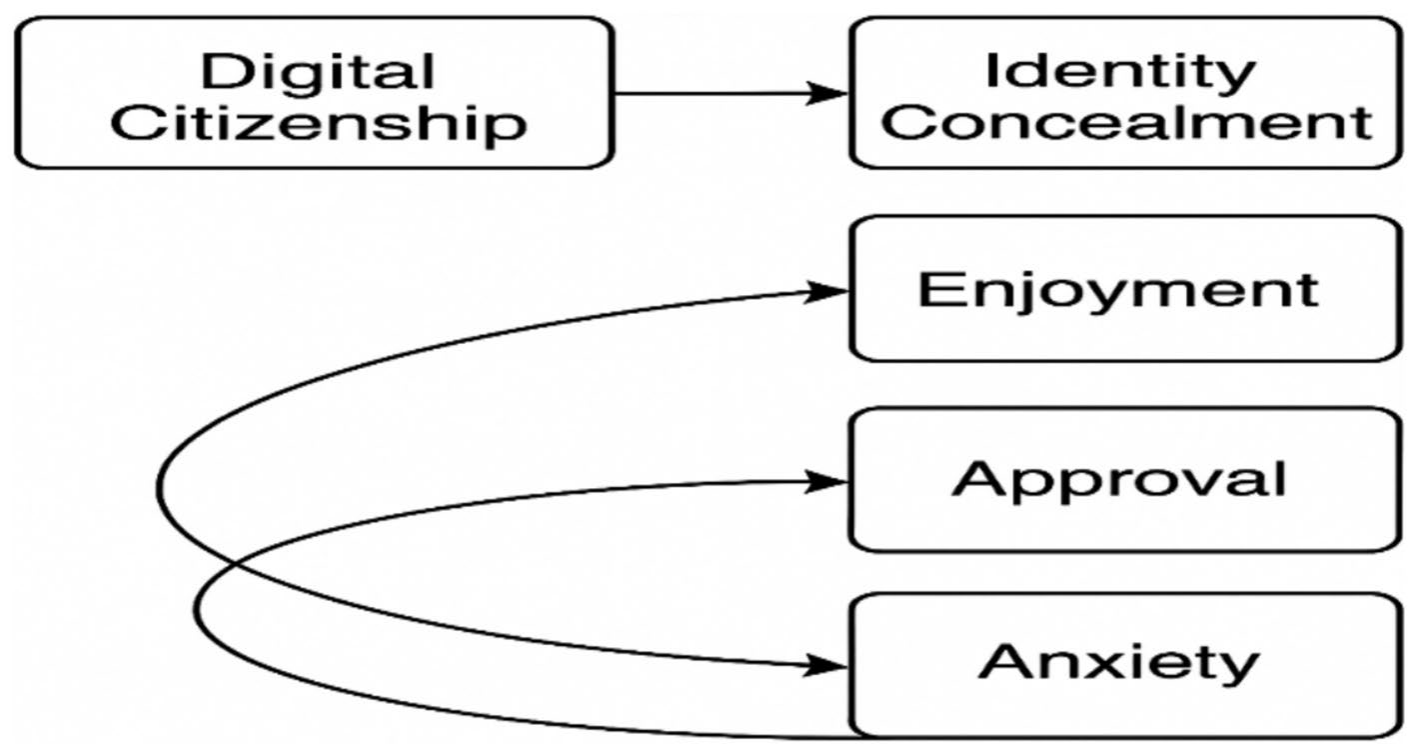
Research model.

Research model: The relational survey model was utilised in this study to examine the relationship between digital citizenship and the levels of cyberbullying exhibited by prospective physical education and sports teachers. Karasar (2015) described the relational survey model as “research models that aim to determine the existence and/or degree of change between two or more variables together.” A correlational screening model was used to investigate the relationship between digital citizenship and cyberbullying levels among physical education and sports teacher candidates. According to Karasar (2011), correlational screening models “aim to determine the existence and/or degree of change between two or more variables.” Path analysis, a type of Structural Equation Modelling (SEM) developed by Wright (1934), was used to examine the predictive relationships between variables. In this model, digital citizenship was considered an exogenous (independent) variable, while the four subscales of cyberbullying attitude – anonymity, enjoyment, approval, and anxiety – were regarded as endogenous (dependent) variables. Path analysis is a methodological framework that facilitates the examination of both direct and indirect effects between observed variables. In this particular context, path analysis was employed to elucidate the predictive relationships between variables. This analysis, which is a type of Structural Equation Modelling, was developed by Wright (1934) to reveal the relationships between observed variables. The theoretical model developed within the scope of the study is presented in Figure 1.

The convenience sampling method employed in this study is recognised as a technique that restricts the representativeness of the population. Convenience sampling is a sampling method that relies on the researcher to select participants who are easily accessible. This is generally preferred due to practical reasons such as time, cost, and resource constraints. Nevertheless, it must be noted that this method may not fully represent the heterogeneous nature of the population, and that the generalisability of the findings may be limited due to differences between the sample and the population. In this context, it is important to note that the results of the study should be considered limited to the university students who participated in the study and should not be directly generalised to the broader student population. In order to ensure the validity of the results, it is essential that researchers interpret the findings with this limitation in mind. Furthermore, it is recommended that more representative sampling methods be employed in future studies.

### 2.2 Population and sample

The population of this study consists of 490 prospective Physical Education and Sports teachers who are enrolled in the Physical Education and Sports Teacher Education Departments in Faculties of Sports Sciences at Ardahan University, Atatürk University and Fırat University in the 2022–2023 academic year. The sample group consists of 323 prospective Physical Education and Sports teachers, 130 females and 193 males, who studied in the Physical Education and Sports Teaching Departments determined by the convenience sampling method (Gratton and Jones, 2010). The descriptive characteristics of the sample group are presented in Table 1.

**Table 1:** Information on demographic characteristics of participants.

Variables	Groups	Frequency (*n*)	Percentage (%)	*X_*
Gender	Male	193	59.8	
	Female	130	40.2	
Age				22.41
Computer usage duration	1–2 years	39	12.1	
	3–5 years	284	87.9	
Internet usage duration	3–5 years	97	30.0	
	6 years+	226	70.0	
Daily internet usage duration	1–2 h	13,9	13.9	
	3–5 h	39,9	39.9	
	6 h+	46,1	46.1	
Internet usage skills	Moderate	173	53.6	
	High	150	46.4	

### 2.3 Data collection tools

The utilisation of data collection tools was enabled by the preparation of an information form, which contained the demographic data of the participants. This form was prepared following consultation with experts in the field.

The Digital Citizenship Scale, a tool developed by İşman and Güngören (2014), was utilised as a data collection instrument in the present study. The 33-item scale is divided into nine subscales: digital communication, digital access, digital literacy, digital security, digital etiquette, digital rights and responsibilities, digital law, digital health and wellness, and digital commerce. A higher score on the digital citizenship scale indicates that digital technology and the internet are utilised more consciously. In the 5-point Likert-type scale, the responses are categorised as follows: “strongly agree” = 5, “agree” = 4, “undecided” = The scale of evaluation comprised three categories: “disagree” (2), “strongly disagree” (1), and “disagree” (3). The five negative items were reverse scored. In this study, the internal consistency of the scale was determined by the Cronbach Alpha coefficient, which yielded a value of 0.92.

The Cyberbullying Attitude Scale developed by Türkoğlu (2013) was used as the other data collection tool in the study. The 42-item scale consists of 4 subscales: identity concealment, enjoyment, approval, and anxiety. An increase in the score obtained from the cyberbullying attitude scale means that the tendency toward cyberbullying also increases. The subscales of the cyberbullying attitude scale include identity concealment, which refers to using digital technology and the Internet by hiding oneself, enjoyment subscale, which refers to enjoying unethical use of digital media and the Internet, approval subscale, which refers to accepting unethical behaviors on digital technology and the Internet, and anxiety subscale, which refers to the uneasiness that some information on digital technology and the Internet may be used by others. The five-point Likert-type scale includes positive and negative statements ranging from “strongly disagree” to “strongly agree.” The answers to the negative statements in the scale were reverse scores as “Strongly agree: 1,” “Agree: 2,” “Partially agree: 3,” “Disagree: 4,” and “Strongly disagree: 5.” In this study, the internal consistency of the scale was determined by the Cronbach Alpha coefficient and found to be 0.90.

### 2.4 Data collection process

The data were obtained through the scales prepared by the researchers by informing the participants face-to-face.

The following detailed revision of the Data Collection Procedure is hereby presented for consideration.

The collection of data was conducted in person, either in a classroom or laboratory setting. Prior to participation, all subjects were provided with both verbal and written information regarding the study, including its objectives, the nature of the data to be collected, and the anticipated duration of participation. The participants were evidently informed that:

Their participation was entirely voluntary.

Participants are at liberty to withdraw from the study at any time without consequence.

The responses provided by these individuals will be kept confidential and anonymous, with only aggregated data being reported.

The collected data will be used solely for research purposes.

In order to mitigate the influence of social desirability bias, participants were encouraged to respond with honesty, and it was emphasised that there were no “right” or “wrong” answers. The participants were assured that their individual responses would not be shared with their instructors or peers, and that their personal identities would not be linked to their responses. The scales were administered in a private, supervised environment to ensure comfort and honesty during completion. Following the provision of this information, participants provided written, informed consent prior to completing the questionnaires.

### 2.5 Data analysis

The statistical analysis of the data obtained from the scales was conducted utilising SPSS 25.0 and AMOS 22.0 package software. Subsequent to this stage, extreme value analyses were evaluated by considering the Mahalanobis distance. Following the implementation of the outlier analysis, the data of 12 participants was excluded from further analysis. The analysis was thus performed on the data of the remaining 323 people. Following the verification of the structure of the scales, the skewness kurtosis values for the normality test were initially examined. Consequently, it was determined that the data demonstrated a normal distribution, as evidenced by the values falling between −1.5 and +1.5, and the Q-Q graph not displaying any deviations from the distribution (Tabachnick and Fidell, 2013). In the course of the present study, the following statistical values were taken into consideration in the testing of the scales: the chi-squared statistic (χ2/df), the root mean square error of approximation (RMSEA), the standardised root mean square residual (SRMR), the comparative fit index (CFI) and the goodness of fit index (GFI). Following this stage, One-Way Multivariate Analysis of Variance (MANOVA) was employed to ascertain whether there was a discrepancy between the scores of the participants according to the gender status variable. In order to conduct a multivariate analysis of variance (MANOVA) analysis, the variance and covariance matrices must be homogeneous. The homogeneity of these matrices was examined using the Levene F test and Box’s M test. Tabachnick et al. (2007) posit that when the assumptions are met as a result of these analyses, Wilks’ Lambda (*λ*) value should be taken into account, and that when the assumptions are not met, Pillai’s Trace value should be taken into account. In order to examine the relationships between the variables in accordance with the hypotheses, Pearson Product Moment Correlation analysis was used. The coefficients obtained as a result of this analysis were evaluated according to Schober et al. (2018) (0.00–0.10, insignificant, 0.10–0.39, weak, 0.40–0.69, moderate, 0.70–0.89, strong, 0.90–1.00: very strong).

Following this stage, an examination was conducted to ascertain the presence of multicollinearity between the variables. This was undertaken by means of Pearson Product Moment Correlation analysis, which yielded values below 0.70 (Tabachnick and Fidell, 2013). The present study examined the predictive role of digital citizenship on identity concealment, enjoyment, approval, and anxiety using Path analysis. The model was based on the total score, with latent variables excluded from the analysis. In the model, digital citizenship, regarded as a single scale, was designated as an exogenous (independent) variable, while identity concealment, enjoyment, approval, and anxiety, which were examined as four distinct subscales, were designated as endogenous (dependent) variables.

## 3 Results

In Table 2, Cronbach’s Alpha, an internal consistency value, ranges from 0 to 1, with increasing values corresponding to enhanced consistency and reliability (Cronbach, 1990). In the present study, the total Alpha value of the Digital Citizenship Scale was found to be 0.924, and the total Alpha value of the Cyberbullying Attitude Scale was found to be 0.900. The normality assumption was tested by examining the skewness and kurtosis values, which were found to be within the range of ± 1.5 (Tabachnick and Fidell, 2013). Within this context, it was assumed that the data obtained from both scales were normally distributed.

**Table 2:** Mean, standard deviation, skewness and kurtosis, and alpha values of the subscales in the study.

Digital citizenship subscales	X_	SD	Alpha	Skewness	Kurtosis
Digital Literacy	20.832	5.037	0.831	−0.531	0.254
Digital Law	16.126	3.923	0.847	−1.115	0.959
Digital Rights and Responsibilities	15.544	3.667	0.856	−0.832	0.347
Digital Health and Wellness	9.427	3.038	0.676	−0.166	−0.405
Digital Communication	14.625	3.805	0.849	−0.622	−0.009
Digital Security	9.795	2.729	0.550	−0.095	−0.466
Digital Access	11.102	2.927	0.839	−0.763	0.389
Digital Etiquette	11.154	2.468	0.378	−0.604	0.603
Digital Commerce	11.145	3.088	0.820	−0.637	−0.088
Total Alpha (Internal Consistency) Value: 0.924					

Cyberbullying attitude subscales	X_	SD	Alpha		
Identity Concealment	21.668	12.410	0.959	1.417	1.047
Enjoyment	17.575	10.367	0.949	1.379	0.827
Approval	48.405	11.618	0.928	−1.130	0.613
Anxiety	33.216	7.411	0.914	−1.176	0.776
Total Alpha (Internal Consistency) Value: 0.900					

This study examined gender-based differences in students’ digital citizenship and digital experience subscales. Multivariate analysis of variance (MANOVA) results revealed no significant effect of gender on all dependent variables (Pillai’s Trace = 0.086, *F* = 2.244, *p* = 0.08). However, univariate analyses of variance (ANOVA) revealed significant differences in certain subscales. Male students scored significantly higher on the digital law and digital rights and responsibilities subscales. These findings suggest that male students may have more developed awareness in these areas. Similarly, male students also scored higher on the approval-seeking and digital anxiety subscales. The difference in digital anxiety level was particularly notable, with a significant, near-medium effect size. However, no significant gender-based differences were found on many subscales such as digital literacy, digital health and well-being, digital communication, security, and access. This suggests that basic digital skills are developed at similar levels among male and female students. The gender difference in the digital etiquette and digital commerce subscales was found to be statistically significant, with a trend favouring male students in these subscales as well. Overall, the findings suggest that gender may influence some digital behaviors and perceptions, but this influence is limited (Table 3).

**Table 3:** MANOVA results of digital citizenship and cyberbullying subscale scores according to gender variable.

Subscales	Gender	*n*	*X_*	SD	*F*	*p*	Eta Square (η2)
Digital Literacy	Female	193	20.9119	4.93156	0.118	0.732	
	Male	130	20.7154	5.20768			
Digital Law	Female	193	15.6373	4.00560	7.620	0.006*	**0.023**
	Male	130	**16.8538**	3.69603			
Digital Rights and Responsibilities	Female	193	15.0777	3.69532	7.947	0.005*	**0.024**
	Male	130	**16.2385**	3.52797			
Digital Health and Wellness	Female	193	9.4715	2.92982	0.101	0.750	
	Male	130	9.3615	3.20369			
Digital Communication	Female	193	14.4819	3.83663	0.681	0.410	
	Male	130	14.8385	3.76397			
Digital Security	Female	193	9.7409	2.72053	0.192	0.661	
	Male	130	9.8769	2.75066			
Digital Access	Female	193	11.1036	2.92967	0.000	0.991	
	Male	130	11.1000	2.93561			
Digital Etiquette	Female	193	10.9378	2.42733	3.736	0.054	
	Male	130	11.4769	2.50338			
Digital Commerce	Female	193	10.8808	2.98979	3.549	0.060	
	Male	130	11.5385	3.20163			
Identity Concealment	Female	193	22.2383	12.35985	1.010	0.316	
	Male	130	20.8231	12.48408			
Enjoyment	Female	193	18.1140	10.19740	1.293	0.256	
	Male	130	16.7769	10.60369			
Approval	Female	193	46.8394	11.89626	8.928	0.003*	**0.027**
	Male	130	**50.7308**	10.82617			
Anxiety	Female	193	31.9275	7.78051	15.149	0.000*	**0.045**
	Male	130	**35.1308**	6.38966			

Box’s M *p* = 0.006; Pillai’s Trace = 0.086; *p* = 0.08; *F* = 2.244; Eta Square (*η*^2^) = 0.086. *p < 0.05: significant. Bold expresses the level of statistical significance (p-value).

The findings indicate an absence of a relationship between the age variable of the participants and the subscales of the digital citizenship scale and the subscales of the cyberbullying attitude scale (*p* > 0.05) (Table 4).

**Table 4:** Pearson correlation test results of digital citizenship and cyberbullying attitude subscale scores according to age variable.

Subscales	Age	
Digital Literacy	r	0.026
	p	0.645
Digital Law	r	0.037
	p	0.506
Digital Rights and Responsibilities	r	0.046
	p	0.410
Digital Health and Wellnes	r	0.018
	p	0.741
Digital Communication	r	−0.041
	p	0.463
Digital Security	r	0.015
	p	0.787
Digital Access	r	−0.013
	p	0.815
Digital Etiquette	r	−0.046
	p	0.414
Digital Commerce	r	−0.019
	p	0.731
Identity Concealment	r	−0.052
	p	0.356
Enjoyment	r	−0.034
	p	0.547
Approval	r	0.020
	p	0.718
Anxiety	r	−0.003
	p	0.956

**Figure 2:**
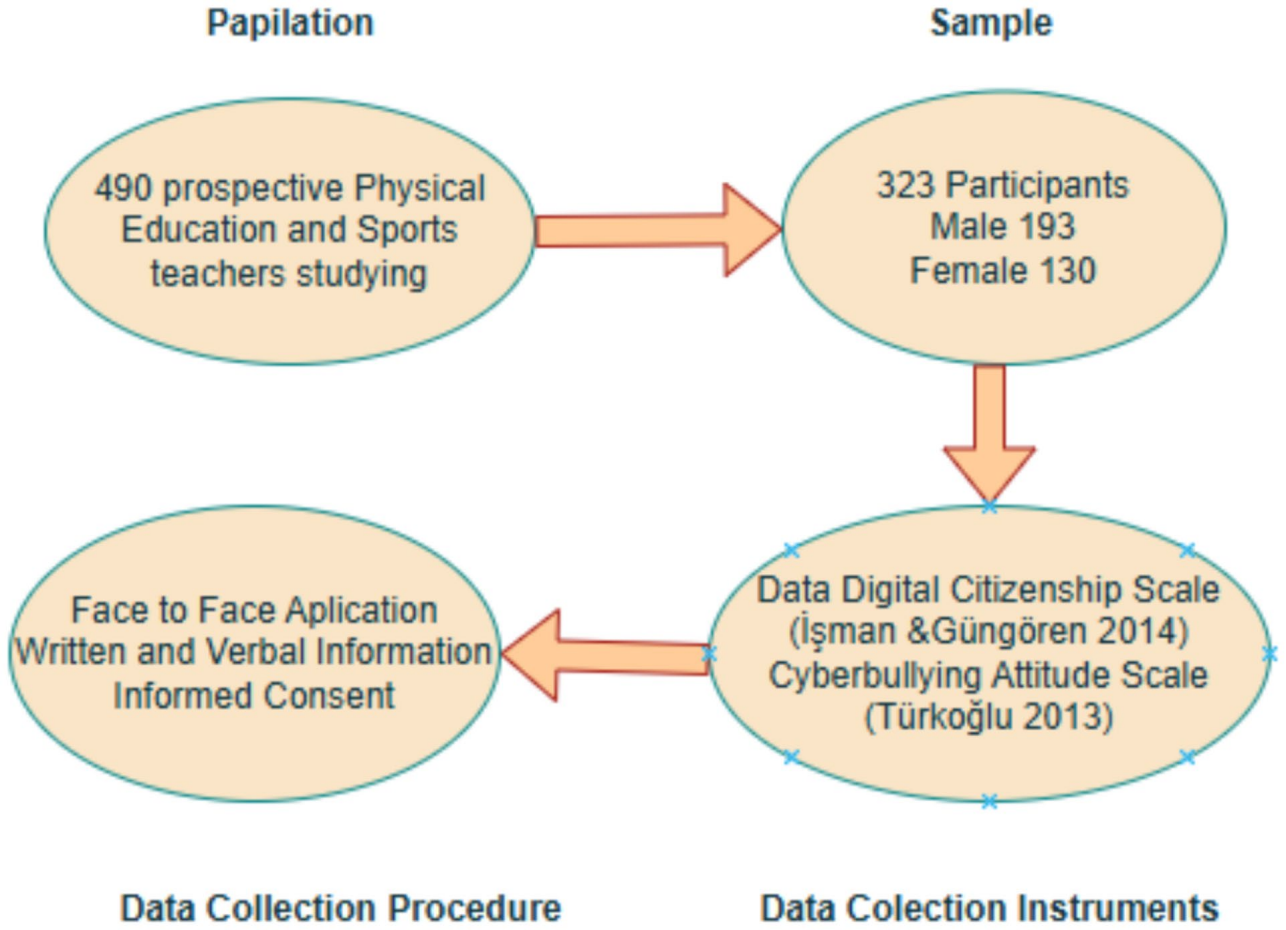
Data collection procedure.

The fit index values for the second level confirmatory factor analysis for the digital citizenship scale are presented in Table 5 [*X*^2^ = 1477.99; df = 483 (*p* < 0.000); *X*^2^/df = 3.06; GFI = 0.77; CFI = 0.77; IFI = 0.80; SRMR = 0.09 and RMSEA = 0.08]. When the ratio of the chi-square value to the degrees of freedom is less than 5, it is indicative of a satisfactory fit between the model and the data (Byrne, 1994; Netemeyer et al., 2003). Root mean square error of approximation (RMSEA) and standardised root mean squared residual (SRMR) values below 0.10 and Goodness of Fit Index (GFI), Comparative Fit Index (CFI), and Incremental Fit Index (IFI) values above 0.90 indicate that the values of the measurement model meet the acceptable fit criteria (Brown, 2006; Kline, 2015). In the present study, the CFI, GFI, and IFI values approximated the critical value of 0.80. When the model is evaluated in its totality, the calculated goodness of fit values demonstrate that the nine-factor structure of the digital citizenship scale is confirmed. Upon examination of the correction indices, it was determined that a substantial enhancement to the model could be realised through the correlation of the errors of variables e8-e10, e26-e27, and e14-e15. In accordance with the assertion posited by Şimşek (2007) that the corrections applied to the indicator variables of a shared latent variable do not result in any issues, the requisite correction was implemented. The path diagram of the second-level Confirmatory Factor Analysis (CFA) analysis of the digital citizenship scale is presented in Figure 2.

**Table 5:** Fit index values of digital citizenship scale according to CFA results.

Scale	*x^2^*	df	*p*	X^2^/df	RMSEA	SRMR	CFI	GFI	IFI
Digital citizenship	1477.99	483	0.000	3.06	0.08	0.9	0.80	0.77	0.80

**Figure 3:**
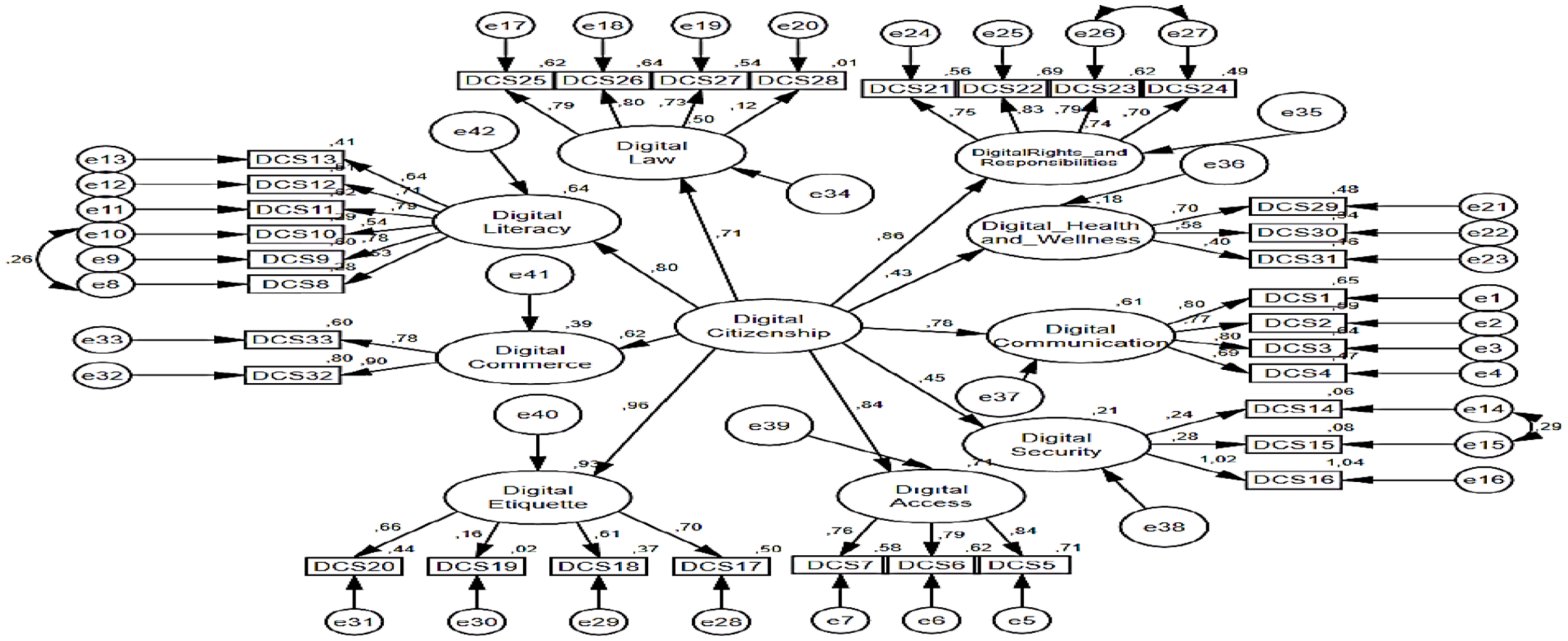
Path diagram of digital citizenship scale second level CFA analysis.

The fit index values related to the first level CFA analysis for the cyberbullying attitude scale are presented in Table 6 [*X*^2^ = 2844.511; df = 809 (*p* < 0.000); *X*^2^/df = 3.51; GFI = 0.68; CFI = 0.84; IFI = 0.84; SRMR = 0.05 and RMSEA = 0.08]. When the ratio of the chi-square value to the degrees of freedom is less than 5, it is indicative of an adequate fit between the model and the data (Byrne, 1994; Netemeyer et al., 2003). The values of RMSEA and SRMR falling below 0.10, and the values of GFI, CFI and IFI exceeding 0.90, are indicative of the measurement model meeting the acceptable fit criteria (Brown, 2006; Kline, 2015). The CFI, GFI, and IFI values were found to be in close proximity to the critical value of 0.80. When the model is evaluated in its totality, the calculated goodness of fit values demonstrate that the nine-factor structure of the digital citizenship scale is confirmed. An examination of the correction indices revealed that a substantial enhancement to the model could be realised through the correlation of the errors of the e13-e14, e24-e25, e2-e3, and e4-e5 variables. In accordance with the assertion posited by Şimşek (2007) that the corrections applied to the indicator variables of a shared latent variable do not result in complications, the requisite correction was implemented. As illustrated in Figure 3, the pathway diagram of the initial level of the CFA analysis of the Cyberbullying Attitude Scale is presented.

**Table 6:** Fit index values of cyberbullying attitude scale according to CFA results.

Scale	*x* ^2^	df	*p*	X^2^/df	RMSEA	SRMR	CFI	GFI	IFI
Cyberbullying	2844.511	809	0.000	3.51	0.8	0.05	0.84	0.68	0.84

In the Path analysis, firstly, it is tested whether the relationships between the variables in the measurement model are significant and it is expected to be verified. In the second stage of the Path, the structural model created based on the theoretical background is tested (Kline, 2015). In the subsequent phase of data analysis, Pearson Product Moment Correlation analysis was employed to assess the relationships between the exogenous variable “Digital Citizenship” and the endogenous variable “Cyberbullying.”

**Figure 4:**
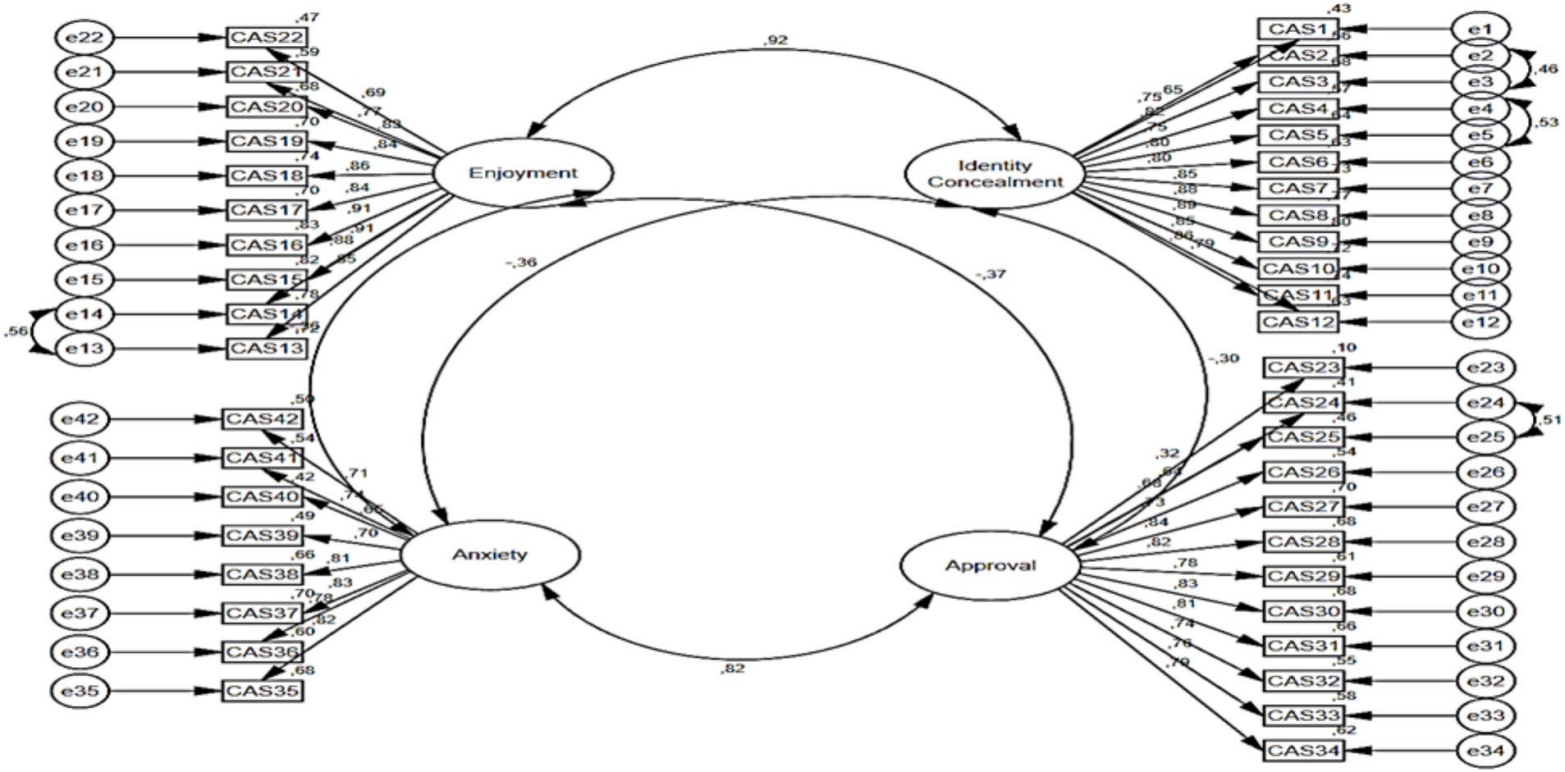
Path diagram of second level CFA analysis of cyberbullying attitude scale.

The Pearson correlation test was conducted in order to ascertain whether there is a significant relationship between the subscales of the digital citizenship scale and the subscales of the cyberbullying attitude scale. The results of this test are presented in Table 7. The findings indicate a low-level positive significant relationship between digital literacy and approval subscales (*r* = 0.113; *p* < 0.05). A low-level negative significant relationship was identified between the digital law subscale and the subscales of identity concealment (*r* = −0.248; *p* < 0.05) and enjoyment (*r*: −0.243; *p* < 0.05). Furthermore, low-level positive significant relationships were identified between the subscales of approval (*r* = 0.195; *p* < 0.05) and anxiety (*r* = 0.196; *p* < 0.05). The findings of the study indicated a low-level negative significant relationship between digital rights and responsibilities and identity concealment (*r* = −0.194; *p* < 0.05) and enjoyment (*r* = −0.212; *p* < 0.05) subscales. In addition, the study revealed a low-level positive significant relationship between approval (*r* = 0.217; *p* < 0.05) and anxiety (*r* = 0.179; *p* < 0.05) subscales. A low-level positive significant relationship was identified between the digital access subscale and the approval subscale (*r* = 0.143; *p* < 0.05). Conversely, a low-level negative significant relationship was identified between the digital commerce subscale and the subscales of identity concealment (*r* = −0.141; *p* < 0.05) and enjoyment (*r* = −0.168; *p* < 0.05). Furthermore, a low-level positive significant relationship was found between the subscales of approval (*r* = 0.142; *p* < 0.05) and anxiety (*r* = 0.168; *p* < 0.05). Subsequent to this stage, the Path analysis was conducted, and the results are presented in Figure 4.

**Table 7:** Pearson correlation analysis of digital citizenship and cyberbullying attitude subscales.

Subscales (*n* = 323)		Identity concealment	Enjoyment	Approval	Anxiety
Digital Literacy	r	−0.027	−0.067	0.113**	0.065
	p	0.631	0.233	**0.042**	0.246
Digital Law	r	−0.248**	−0.243**	0.195**	0.196**
	p	**0.000**	**0.000**	**0.000**	**0.000**
Digital Rights and Responsibilities	r	−0.194**	−0.212**	0.217**	0.179**
	p	**0.000**	**0.000**	**0.000**	**0.001**
Digital Health and Wellness	r	0.079	0.099	−0.042	0.024
	p	0.157	0.075	0.447	0.667
Digital Communication	r	−0.075	−0.062	0.076	0.030
	p	0.180	0.268	0.175	0.587
Digital Security	r	0.106	0.080	−0.056	−0.031
	p	0.057	0.153	0.315	0.585
Digital Access	r	−0.034	−0.072	0.143**	0.057
	p	0.548	0.198	**0.010**	0.304
Digital Etiquette	r	−0.070	−0.076	0.104	0.062
	p	0.207	0.175	0.062	0.267
Digital Commerce	r	−0.141**	−0.168**	0.142**	0.168**
	p	**0.011**	**0.002**	**0.010**	**0.002**

**p < 0.001: stronger significance. *p < 0.05: significant. Correlation Coefficient (r); Significance Level (p).

**Figure 5:**
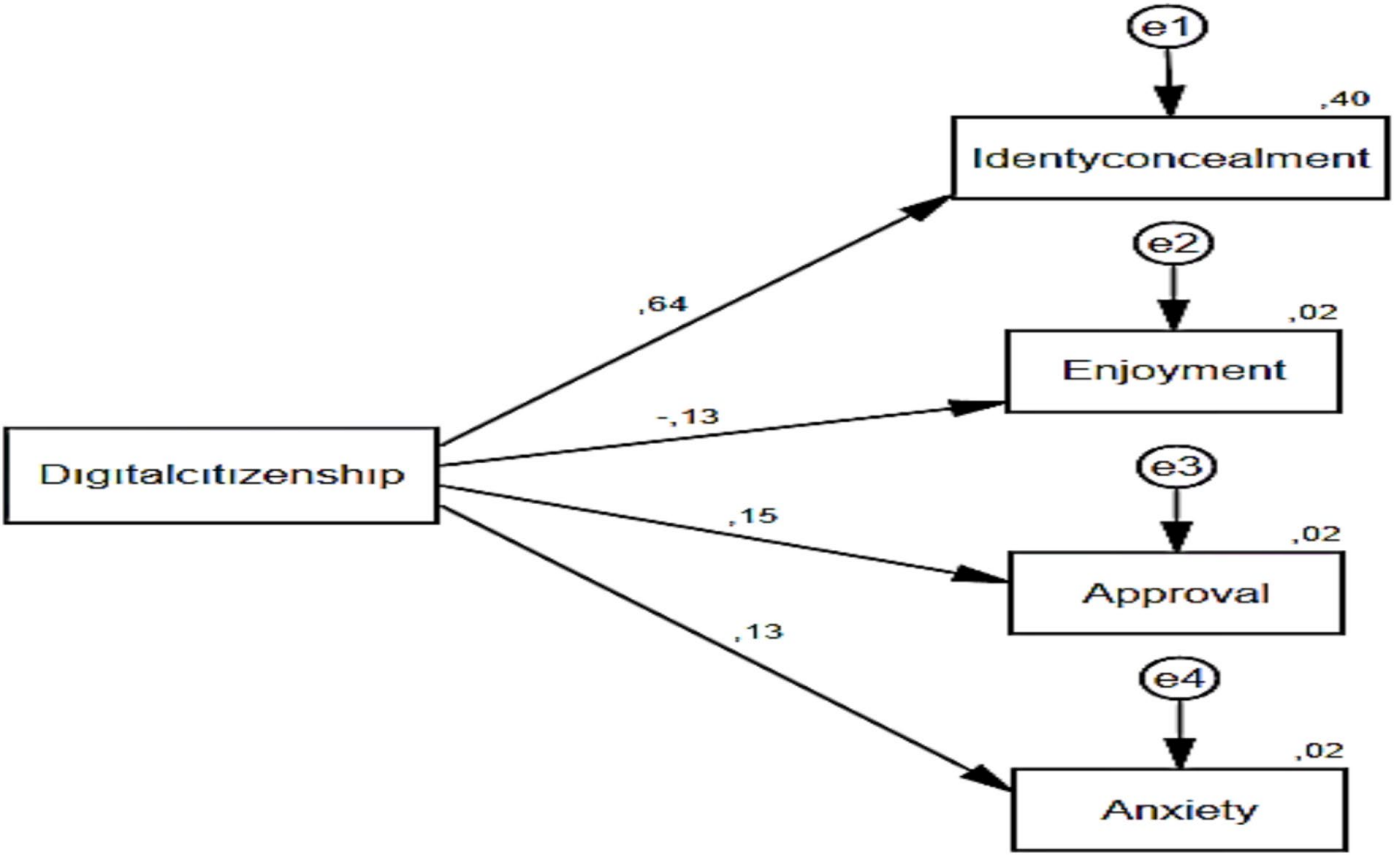
Path analysis for the prediction of identity concealment, enjoyment, approval, and anxiety subscales.

A thorough examination of the values associated with the model presented in Table 8 revealed that digital citizenship exhibited a substantial predictive capacity for identity concealment, accounting for 40% of the observed variance (*β* = 0.635; *R*^2^ = 0.40; *p* < 0.05). The findings of the study indicated that digital citizenship exhibited a substantial predictive capacity for arbitrariness, accounting for 2% of the observed variance (*β* = −0.129; *R*^2^ = 0.02; *p* < 0.05). The findings of the study indicated that digital citizenship exhibited a substantial predictive capacity for approval, accounting for 2% of the observed variance (*β* = 0.155; *R*^2^ = 0.02; *p* < 0.05). Furthermore, the findings of the study indicated that digital citizenship exhibited a substantial predictive capacity for anxiety levels, accounting for 2% of the observed variance (*β* = 0.129; *R*^2^ = 0.02; *p* < 0.05) (Figure 5).

**Table 8:** Path analysis results for digital citizenship scale and identity concealment, enjoyment, approval, and anxiety subscales.

Model	βeta	S.E	C.R.	*p*	*R* ^2^
Digital Citizenship → Identity Concealment	0.635	51.17	–	***	0.40
Digital Citizenship → Enjoyment	−0.129	8.30	−2.326	***	0.02
Digital Citizenship → Approval	0.155	10.35	2.815	***	0.02
Digital Citizenship → Anxiety	0.129	4.24	2.326	***	0.02

***p < 0.001: very strong significance.

## 4 Discussion

A MANOVA analysis was conducted on the digital citizenship and cyberbullying attitude subscales, with the gender variable serving as the independent variable. The results of this analysis revealed a significant difference in the integrated effect. A subsequent examination of the results between the subscales reveals a significant difference in the digital law and digital rights and responsibilities subscales. Upon analysis of the mean scores, it is evident that male participants demonstrate higher mean scores in comparison to their female counterparts. A significant discrepancy was also observed between approval and anxiety levels. A close examination of the mean scores reveals that male participants demonstrate higher averages in comparison to their female counterparts. A study conducted on a sample of university students studying communication sciences revealed that males exhibited a higher propensity for cyberbullying attitudes in comparison to their female counterparts (Karadağ and Banar, 2022). In the context of studies conducted with university students, it has been observed that cyberbullying tendencies are more prevalent among males (Arıcak, 2009; Akbulut and Erişti, 2011; Dilmaç, 2009; İğdeli, 2018). In a further study conducted among high school students, it was found that the cyberbullying tendencies of male students were higher than those of female students, and that the difference between the two groups was significant (Nazik et al., 2020). It was asserted that university students are becoming increasingly susceptible to cyberbullying and cyberharassment. In addition to risk factors and negative experiences, conscientious online behaviour and support seeking play a protective role. It was asserted that academic institutions should assume an active role in the implementation of preventive interventions, utilising evidence-based programmes (Bussu et al., 2024). While the increased use of the internet and social media by university students renders them more vulnerable to cyberbullying, it has been reported that the role of personal, psychological, and environmental factors is critical in the development of policies and strategies to prevent cyberbullying (Shaikh et al., 2020). Abaido (2020) determined that university students in Arab communities are frequently subjected to cyberbullying on social media platforms, and that reporting of these incidents is limited due to cultural and social constraints. Furthermore, he emphasised the significance of awareness-raising programmes, stringent legal regulations, and proactive measures. The perpetuation of cyberbullying is influenced by a combination of factors, including the perception of online disinhibition, which is itself influenced by gender. Research indicates that self-control is a critical buffer against the intention to perpetrate cyberbullying (Wong et al., 2018). As demonstrated by Mishna et al. (2020), there is a tendency to target girls and attribute blame to them for gender-based and sexualised bullying, while boys are frequently rendered invisible. This finding suggests that bullying may be influenced by gender norms and stereotypes, potentially resulting in girls anticipating inequality and aggression during the socialisation process. Marr and Duell’s (2020) study demonstrates that the judgments made in cases of cyberbullying vary according to the gender of the cyberbully, the victim, and the evaluator. This finding suggests that gender norms and biases influence perceptions of cyberbullying and fairness judgments, emphasising the necessity for gender-sensitive educational and policy approaches. As demonstrated by Zhong et al. (2021), a multitude of factors, including personal history, gender, personality, and digital citizenship level, have been shown to exert a significant influence on the prevalence of cyberbullying and victimization among university students. It was asserted that while digital literacy and adherence to internet etiquette play a protective role, online habits and internet addiction increase the risk, thereby highlighting the importance of multidimensional cyberbullying prevention strategies. In the study conducted by Peled (2019), it was observed that undergraduate students are frequently exposed to instances of cyberbullying, particularly through the medium of instant messaging. This phenomenon has been found to exert a detrimental effect on the academic, social and emotional development of the affected students. While factors such as gender, religion, and sexual orientation are important in understanding the effects, the need for specific attention to this population in future research has been emphasised. Consequently, the present study corroborates the findings of preceding research in the relevant literature.

Conduct of the Pearson correlation test yielded a low-level positive significant relationship between the digital literacy and approval subscales of the digital citizenship scale and the cyberbullying attitude scale subscales. A low-level negative significant relationship was identified between the digital law subscale and the identity concealment and enjoyment subscales, and a low-level positive significant relationship was identified between the approval and anxiety subscales. A low-level negative significant relationship was identified between the digital rights and responsibilities subscale and the identity concealment and enjoyment subscales. Conversely, a low-level positive significant relationship was identified between the approval subscale and anxiety subscales. A substantial negative correlation has been demonstrated between levels of digital citizenship and the propensity for cyberbullying among university students, according to the findings of recent research. Research has indicated that higher digital citizenship, defined as the capacity to comprehend and adhere to internet etiquette, digital legislation, and responsible online conduct, is correlated with a decline in cyberbullying behaviour. Students who possess a robust comprehension of digital ethics and legality demonstrate a reduced propensity to engage in cyberbullying behaviours. Conversely, students grappling with internet addiction or exhibiting deficient digital communication skills are susceptible to an elevated risk of such behaviours. However, the present study found that digital citizenship was only significantly correlated with perpetrating cyberbullying, rather than being a victim of it (Zhong et al., 2021; Dunaway and Macharia, 2021). Hassan et al. (2023) stated that there is a significant correlation between cyberbullying and social media addiction among law students. The researchers concluded that, while anonymity facilitates these behaviours, gender and academic level differences do not have an effect. Martínez-Monteagudo et al. (2020) demonstrate a substantial correlation between cyberbullying and suicidal ideation, as well as elevated anxiety, depression and stress levels. This underscores the necessity for efficacious interventions within university settings. Karakuş and Turan’s (2022) study, titled “Examining the Relationship between Adults’ Cyber Bullying Behaviours and Digital Citizenship Skills,” and Kaptangil and Çalışır’s (2023) study, titled “Moderating Effect of Alexitimia on the Relationship between Digital Citizenship and Cyber Bullying,” both found low-level negative significant relationships. Consequently, the present study corroborates the findings of preceding research in the relevant literature.

A low-level positive significant relationship was identified between the digital access subscale and the approval subscale. A low-level negative significant relationship was identified between the digital commerce subscale and the identity concealment and enjoyment subscales, and a low-level positive significant relationship was identified between the approval and anxiety subscales. Concurrent with this study, the correlation results demonstrate a relationship between digital citizenship and the cyberbullying attitudes of prospective primary school teachers. The findings reveal a significant relationship between digital citizenship levels and cyberbullying tendencies (Çiftçi and Sakallı, 2016).

A subsequent examination of the values associated with the results of the Path analysis of the digital citizenship scale and its subscales (identity concealment, enjoyment, approval, and anxiety) revealed a significant prediction of the subscales by digital citizenship, with a total variance of 46%. Within this scope, in the study titled “Examining the Relationship between Adults’ Cyber Bullying Behaviors and Digital Citizenship Skills” by Karakuş and Turan (2022), it was determined that digital citizenship predicted cyberbullying by 18%, which is in parallel with the current study. The present study makes a contribution to the extant theoretical framework by demonstrating a strong correlation between advanced digital competencies and ethical online behaviour. The findings demonstrate a clear correlation between digital citizenship dimensions such as digital literacy, digital rights and responsibilities, and digital law, and attitudes toward cyberbullying. The findings of this study indicate that educational interventions designed to cultivate digital citizenship may prove efficacious in diminishing cyberbullying propensities among students. Furthermore, these results emphasise the importance of incorporating a gender-based analysis into research and intervention strategies to comprehensively address this pressing issue.

### 4.1 Limitations

The study’s findings are limited to physical education preservice teachers from three universities, which limits the generalizability of the results. The authors did not discuss the potential extension of these findings to preservice teachers from other academic fields or to broader groups of preservice teachers. It is recommended that future research include participants from a more extensive range of universities and educational disciplines, with a view to enhancing the applicability of the results. Furthermore, the utilisation of random or stratified sampling methodologies has the potential to enhance the representativeness of the study sample and provide more robust evidence regarding the relationship between digital citizenship and cyberbullying attitudes across diverse groups of preservice teachers.

## 5 Conclusion

This study underscores the correlation between digital citizenship levels and the attitudes toward cyberbullying of prospective physical education and sports teachers, emphasising that digitalisation, while conferring numerous benefits across various aspects of life, concomitantly carries risks when not managed responsibly. The findings emphasise the importance of integrating digital citizenship education into teacher training programmes, with the aim of raising awareness and equipping future educators with the skills to prevent and address cyberbullying in educational settings.

From an academic perspective, the results contribute to the growing body of literature on digital citizenship and cyberbullying by drawing attention to the role of teacher candidates, a group that has been overlooked in related studies. In practice, the findings provide a basis for the development of targeted educational policies and institutional strategies to foster safe and responsible technology use among university students.

Whilst the present study is constrained in its scope to prospective physical education and sports teachers, further research incorporating students from a range of academic disciplines and universities could enhance our comprehension of digital citizenship and cyberbullying. The utilisation of comparative approaches in this manner has the potential to enhance generalizability and to propose alternative solutions to challenges that are becoming increasingly prevalent in professional contexts that are becoming increasingly digitised. An examination of the relationship between university students’ digital citizenship levels and their attitudes toward cyberbullying suggests that the development of skills such as digital literacy, digital rights and responsibilities, and digital law can play a protective role in reducing cyberbullying behaviour. From an academic standpoint, the study makes a substantial contribution to the existing literature by demonstrating that digital citizenship is a significant predictor of online behaviour. The findings indicate that the integration of digital citizenship training into university curricula and teacher training programmes can serve as an effective strategy for curbing cyberbullying and promoting responsible online interaction among students.

### 5.1 Recommendations

A more representative sampling is required. In order to enhance the generalisability of the study’s findings, it is recommended that a more substantial and representative sample be obtained, encompassing students from a variety of university departments and diverse demographic groups.

Comparative Studies: Conducting comparative studies that examine the relationships between digital citizenship levels and cyberbullying attitudes across different populations has the potential to broaden the scope and application of the findings.

This text is intended for educational professionals. Activities, workshops, and lesson plans should be developed to cultivate students’ digital citizenship skills; these programs should include online ethics, responsible sharing, and strategies for dealing with cyberbullying.

The following text is intended for students. It is imperative that policy guidelines and rules for cyberbullying budgets are established at the school and university levels. Furthermore, policies, reporting, and centralised campaigns to prevent this funding should be developed and implemented.

## Data Availability

The raw data supporting the conclusions of this article will be made available by the authors, without undue reservation.
